# Preclinical and Clinical Antioxidant Effects of Natural Compounds against Oxidative Stress-Induced Epigenetic Instability in Tumor Cells

**DOI:** 10.3390/antiox10101553

**Published:** 2021-09-29

**Authors:** Abdelhakim Bouyahya, Naoual El Menyiy, Loubna Oumeslakht, Aicha El Allam, Abdelaali Balahbib, Abdur Rauf, Naveed Muhammad, Elena Kuznetsova, Marina Derkho, Muthu Thiruvengadam, Mohammad Ali Shariati, Nasreddine El Omari

**Affiliations:** 1 Laboratory of Human Pathologies Biology, Department of Biology, Faculty of Sciences, and Genomic Center of Human Pathologies, Faculty of Medicine and Pharmacy, Mohammed V University in Rabat, 4 Avenue Ibn Batouta, Rabat P.O. Box 1014, Morocco; boyahyaa-90@hotmail.fr (A.B.); elallamaicha@gmail.com (A.E.A.); 2 Department of Biology, Faculty of Science, University Sidi Mohamed Ben Abdellah, P.O. Box 1796 Fez 30 000, Morocco; Nawal.ELMENYIY@usmba.ac.ma; 3 Institute of Biological Sciences (ISSB-P), Mohammed VI Polytechnic University (UM6P), Benguerir 43150, Morocco; loubnaoumeslakht@gmail.com; 4 Laboratory of Biodiversity, Ecology and Genome, Faculty of Sciences, Mohammed V University, Rabat 4 Avenue Ibn Batouta, Rabat P.O. Box 1014, Morocco; balahbib.abdo@gmail.com; 5 Department of Chemistry, University of Swabi, Anbar, Khyber Pakhtunkhwa, Swabi 23430, Pakistan; mashaljcs@yahoo.com; 6 Department of Pharmacy, Abdul Wali Khan University Mardan, Khyber Pakhtunkhwa, Mardan 23200, Pakistan; drnaveedrph@gmail.com; 7 Department of Industrial Chemistry and Biotechnology, Orel State University named after I.S. Turgenev, 95 Komsomolskaya St., 302026 Orel, Russia; elkuznetcova@ya.ru; 8 Institute of Veterinary Medicine, South-Urals State Agrarian University, 13 Gagarin St., Chelyabinsk Region, 457100 Troitsk, Russia; derkho2010@ya.ru; 9 Department of Crop Science, College of Sanghuh Life Science, Konkuk University, Seoul 05029, Korea; 10 Department of Scientific Research, K.G. Razumovsky Moscow State University of Technologies and Management (The First Cossack University), 73, Zemlyanoy Val St., 109004 Moscow, Russia; shariatymohammadali@gmail.com; 11 Laboratory of Histology, Embryology and Cytogenetic, Faculty of Medicine and Pharmacy, Mohammed V University in Rabat, 19 Rue Tarik Ibnou Ziad, Rabat P.O. Box 9154, Morocco; nasrelomari@gmail.com

**Keywords:** oxidative stress, cancer, epigenetic, bioactive compounds

## Abstract

ROS (reactive oxygen species) are produced via the noncomplete reduction in molecular oxygen in the mitochondria of higher organisms. The produced ROS are placed in various cell compartments, such as the mitochondria, cytoplasm, and endoplasmic reticulum. In general, there is an equilibrium between the synthesis of ROS and their reduction by the natural antioxidant defense system, called the redox system. Therefore, when this balance is upset, the excess ROS production can affect different macromolecules, such as proteins, lipids, nucleic acids, and sugars, which can lead to an electronic imbalance than oxidation of these macromolecules. Recently, it has also been shown that ROS produced at the cellular level can affect different signaling pathways that participate in the stimulation of transcription factors linked to cell proliferation and, consequently, to the carcinogenesis process. Indeed, ROS can activate the pathway of tyrosine kinase, MAP kinase, IKK, NF-KB, phosphoinositol 3 phosphate, and hypoxia-inducible factor (HIF). The activation of these signaling pathways directly contributes to the accelerated proliferation process and, as a result, the appearance of cancer. In addition, the use of antioxidants, especially natural ones, is now a major issue in the approach to cancer prevention. Some natural molecules, especially phytochemicals isolated from medicinal plants, have now shown interesting preclinical and clinical results.

## 1. Introduction

Oxygen molecules are central molecules that live in cells and are responsible for the oxidation of organic matter. This molecule is considered as a final electron acceptor during cellular respiration in higher organisms of eukaryotic cells. The final oxidation reaction takes place between the protons generated during the oxidation of organic matter and the molecular oxygen located at the mitochondrial level. This reaction results in the formation of H_2_O as the final product of cellular respiration; however, intermediate reactions can occur during this oxygen reduction, and they can give certain reactive oxygen molecules, for example, hypochlorous acid (HOCL), singlet oxygen (^1^O_2_), hydrogen peroxide (H_2_O_2_), and lipid peroxide (LOOH), hydroxyl radical (-OH), alkoxy radical (RO-), superoxide anion (O_2_-), and hydroperoxyl radical (HO_2_-) [1]. In the homeostasis equilibrium, the production of ROS is controlled by a natural antioxidant system constituting some endogenous enzymes that have the capacity to eliminate or reduce these free radicals. These enzymes include catalase (CAT), glutathione peroxidase (GPx), glutathione reductase (GR), and superoxide dismutase (SOD). An imbalance in the respiratory chain and an excessive production of ROS with a low antioxidant capacity have a detrimental impact on many macromolecules. In fact, ROS can oxidize certain macromolecules, such as proteins and nucleic acids. They can also peroxidize lipids, which leads to oxidation and, therefore, an electronic imbalance of these vital molecules [1].

The ROS generated during this reduction in molecular oxygen are involved in several pathologies via many mechanisms: directly, by their involvement in instability in macromolecules in particular oxidation and, consequently, mutation of nucleic acids (DNA), and, indirectly, because ROS can also induce intracellular signaling via which they can activate certain phosphorylation pathways and deactivate others. Indeed, the production of ROS has been rubbished as an indicator of cell proliferation, as most of these reactive species have a positive action on the signaling pathways that stimulate cell proliferation and a negative action on the proteins that control cell division. Via these mechanisms, ROS can induce deregulation of gene expression, since they can change gene expression via a disruption of essentially epigenetic pathways (stimulating transcription factors responsible for cell proliferation and inhibiting others responsible for cell cycle control). This epigenetic instability essentially translates into the ectopic expression of oncogenes and antioncogenes, which favors the process of cancerization. This epigenetic instability can, with time, in one way or another, generate a genetic instability via the induction of the mutation, including those affecting oncogenes (dominant mutation, advantageous) and antioncogenes mutation (deleterious mutation, disadvantageous). In addition, nowadays it appears that the treatment of some cancers must pass through the inhibition of the oxidative process and the process of epigenetic deregulation [2]. Antioxidants that can prevent the production of ROS or possibly block the action of these reactive oxygen species are a major therapeutic way to reinforce the anticancer treatments that exist today.

The use of antioxidants to treat diseases is ancient history in medicine; many antioxidants today are used for the medication of various diseases, in particular, inflammatory diseases (use of vitamin C). For the treatment of cancer, the use of targeted antioxidants (antioxidants that specifically target the production or action of ROS) cannot give direct results for the reason that the pathways of action of ROS are diverse and their mechanisms of action are poorly explained; in this sense, the use of natural antioxidants (via food) could play a very significant role in the prevention of diseases related to the braid, in particular, cancer. Indeed, currently, many preclinical and clinical investigations have shown that some natural substances, in particular, those present in medicinal food plants, have very remarkable antioxidant properties [3].

Therefore, the use of these medicinal food plants could play an important role in implementing natural antioxidants to prevent the appearance of various cancers, but also to use them as a therapeutic agent combined with conventional chemotherapy. The aim of our work is to explain molecularly of the relationship between the production of ROS at the cellular level and the defense system that controls this production, as well as to demonstrate how ROS are directly or indirectly involved in the process of carcinogenesis, and, finally, to propose bioactive molecules from traditional medicine. As an antioxidant agent for the prevention and therapy of several highly oxidation-related cancers.

## 2. Oxidative Stress Signaling Pathways

### 2.1. ROS

Oxygen is a vital gas that is crucial for the production of energy in aerobic organisms, since it is the terminal acceptor of electrons during respiration. It has one unpaired electron in each of its two spins and it is considered as a free di-radical that has low reactivity. Additional energy from external (e.g., ionizing irradiations) or internal sources (e.g., electron transport chains) converts oxygen to a more reactive state, inducing the transfer of its electron between donors and acceptors through redox reactions, which generate ROS. ROS include nonradical molecules, such as HOCL, ^1^O_2_, H_2_O_2_, and LOOH, and free radicals that have one or more unpaired electrons, such as O_2_^•−^, RO^•^, OH, HO_2_^•^, etc. [1].

ROS can be generated from exogenous and endogenous sources. Endogenous sources include the immune system, ischemia, infection, cancer, psychological stress, aging, etc. [4]. Exogenous sources include ionizing radiations, nonionizing radiations (e.g., ultraviolet A, B, or C) [5], pollutants, drugs, xenobiotics (e.g., pesticides, toxins, and herbicides), chemicals (e.g., heavy metals, chemotherapy, and alcohol), and food [6].

ROS are delivered in several cellular compartments, including the plasma membrane, peroxisomes, endoplasmic reticulum, and mitochondria. Their formation is mediated by different enzymatic systems, including nicotinamide adenine dinucleotide phosphate (NADPH) oxidase (NOX) [7], cytochrome C oxidase (CCO) of the mitochondrial electron transport chain [8], lipoxygenase [9], xanthine oxidase [10], cyclooxygenase, cytochrome P450 [11], monooxygenase, and nitric oxide synthase [12].

**Figure 1:**
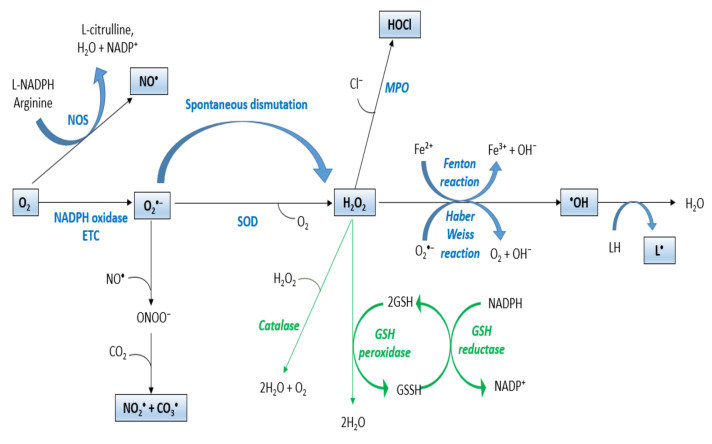
ROS production. ETC: electron transport chain; SOD: superoxide dismutase; MPO: myeloperoxidase; NO^•^: nitric oxide; NOS: nitric oxide synthase; ONOO⁻: peroxynitrite; O_2_^•−^: superoxide anion; H_2_O_2:_ hydrogen peroxide; ^•^OH: hydroxyl radical; NADP: nicotinamide adenine dinucleotide phosphate; HOCL: hypochlorous acid; GSH: glutathione; LH: unsaturated lipid; L^•^: lipid radical.

Adding an electron to an oxygen spin results in the production of O_2_^•−^ with a single unpaired electron and a negative charge. This reaction can be mediated by different enzymes, such as the NADPH oxidase family that comprises seven enzymes, including Nox1–5 and Duox1–2. These enzymes transfer electrons across membranes onto oxygen to generate O_2_^•−^ [13]. O_2_^•−^ can further be reduced to H_2_O_2_ by spontaneous dismutation or by SOD, which are essential enzymes required for the control and prevention of the formation of high levels of ROS [14]. O_2_^•−^ may also interact with peroxynitrite (ONOO^−^) and nitric oxide (NO^•^). Oxidation of l-arginine to l-citrulline can synthesize NO^•^ by NOS using NADPH and O_2_ as co-substrates [15] (Figure 1).

H_2_O_2_, a nonradical molecule, can be reduced by multiple oxidases, such as myeloperoxidase. The reaction of H_2_O_2_ is catalyzed with chloride ions (Cl) by this lysosomal enzyme to form hypochlorous acid (HOCl) in neutrophils and monocytes, which allow host defense and pathogen degradation [16]. In the presence of metal ions, it can also be reduced to ^•^OH by Fenton or Haber–Weiss reactions [17]. These -OH are highly reactive and have an extremely short half-life of 10^−9^ s. They can react rapidly with any oxidizable molecule that they encounter [18] (Figure 1). For instance, they can abstract H atoms from unsaturated lipids (LH) and initiate lipid peroxidation that induces oxidative degradation of lipids, resulting in cell damage [19].

### 2.2. Redox Homeostasis

ROS are required for organism survival since they play crucial functions in various physiological processes, such as apoptosis [20], immune response [21], differentiation [22], regulation of signaling pathways [23], and reproduction [24]. On the other hand, ROS can be signaling molecules and be involved in the inhibition or induction of cell proliferation, apoptosis, and necrosis [25]. The human organism possesses several antioxidant mechanisms that can inhibit the uncontrolled production of free radicals and their reaction with biological structures, or modulate and counteract their effects in order to maintain redox homeostasis. These mechanisms include different scavengers, such as hydrophilic scavengers (ascorbate and glutathione) that are found in mitochondria, cytosolic, and nuclear aqueous compartments. Ergothioneine scavengers and hydrophobic scavengers include vitamin E and carotenoids (β-carotene), which inhibit lipid peroxidation in cell membranes [26]. There are also different enzymatic proteins, such as SOD [27], CAT [28], and GPx [29], having a key role in the protection against the malignant effects of oxidative stress. For instance, SOD catalyzes the superoxide anion dismutation and produces H_2_O_2_ and O_2_ [27]. CAT is an enzyme that is located in the peroxisome. It catalyzes the reduction in H_2_O_2_ to H_2_O and O_2_ when H_2_O_2_ is present at high titers in the cell [28]. GPx is an enzyme that is located in the cell cytoplasm and mitochondria. It catalyzes the reduction in H_2_O to H_2_O_2_ by the oxidation of reduced GSH to GSSH [29]. GR is a cytosolic protein that restores intracellular GSH by reducing glutathione disulfide (GSSG) using NADPH [30] (Figure 1).

Redox homeostasis imbalance with enhanced ROS production and reduced antioxidant activity can damage cells and tissues and lead to various pathologies. In addition, the abnormal accumulation of ROS can alter the structural and functional properties of carbohydrates, nucleic acids, lipids, and proteins [1]. Free radicals can react with amino acids and induce cross-linking, aggregations, and structural disturbance in proteins. They can also directly damage DNA by creating cross-links and altering nucleotide bases and deoxyribose sugars [1]. Hydroxyl radicals can induce lipid peroxidation, which damages the membrane structure and alters its fluidity and function [31]. Radicals can also oxidize mono and polysaccharides and induce their polymerization [1]. These alterations can lead to various pathologies, including cancer.

## 3. ROS Signaling Pathways in Cancer

**Figure 2:**
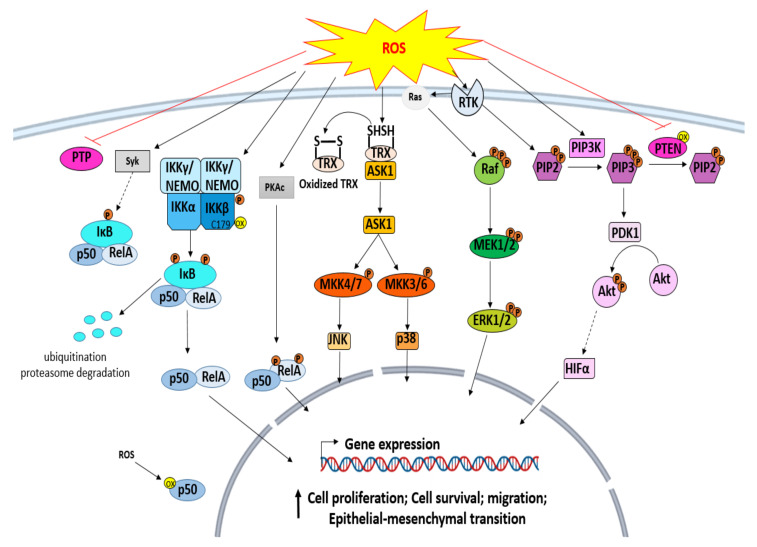
Oxidative-stress-induced signaling pathways in cancer.

ROS are found to be expressed at high levels in various cancer cells. They are highly implicated in carcinogenesis and can favor tumor progression directly by causing DNA damage, or indirectly by the alteration of different signaling pathways implicated in the cell cycle, cell proliferation, cell survival, apoptosis, etc (Figure 2). On signaling pathways, the most significant effects of oxidative stress were noted in MAP kinase, phosphoinositide 3-kinase (PI3K), NF-κB pathways, and HIF-1α that is also activated [32].

### 3.1. ROS and Protein Tyrosine Kinase (PTK)

Protein phosphorylation is affected by PTKs by catalyzing, in protein substrates, the transfer of phosphate from ATP to tyrosine residues. They play a vital role in intracellular signaling, and their catalytic function can be regulated by other different PTKs or protein tyrosine phosphatases (PTPs). This regulation can also be modulated by environmental chemicals or oxidative stress. ROS upregulate the catalytic activities of PTKs through the inactivation of PTPs. They can also directly oxidize SH groups of specific conserved cysteine residues on PTKs or produce disulfide-bonded dimers on PTK proteins [33]. Furthermore, the activation of the cell death or cell cycle depends on the generated ROS levels. Low levels of ROS may lead to cell cycle activation via the upregulation of PTKs, including MAPK proteins, such as serine/threonine kinases, ERK, and transcription factors. Slightly increased levels of ROS induce death through a cascade of signals, started by the activation of Ask1, a member of the MAPK family that activates different signaling pathways, including Bax/BH3, JNK/p35 MAPK, caspases, and cytochrome C. High levels of ROS induce necrosis by damaging mitochondria [33].

### 3.2. Mitogen-Activated Protein Kinases (MAPKs) Signaling Pathway

MAPKs, a family of serine–threonine protein kinases, include ERKs, stress-activated MAPKs, JNKs, and p38 MAPKs. These molecules transduce signals from the cell surface to the nucleus through a cascade of phosphorylation on their threonine and tyrosine residues. They also regulate invasion, apoptosis, differentiation, and cell proliferation [34]. The deregulation of the MAPK signaling pathway is highly involved in human cancer, and oxidative stress is among the crucial factors that lead to this deregulation and induce carcinogenesis. The high levels of ROS can activate these kinases and promote cell migration, proliferation, survival, and epithelial–mesenchymal transition [35]. ROS may directly oxidize tyrosine kinase receptors, which induce activation of Ras-GTPase. Ras phosphorylates Raf, a MAP3K that activates, in turn, ERK via MAP/ERK kinase (MEK) [34]. ROS also oxidize thioredoxin, which dissociates from ASK-1, a MAP3K, and, therefore, ASK-1 activates MKK3, MKK4, MKK6, and MKK7. Moreover, MKK3 and MKK6 phosphorylate and activate p28 MAPK, while MKK4 and MKK7 phosphorylate JNK protein, which is involved in cancer cell apoptosis [34].

### 3.3. ROS and IκB Kinase (IKK)/NF-κB Pathway

NF-κB, a transcription factor regulating the expression of various genes, is involved in various physiological processes, including response to stress, cell survival, immune defense, etc. In the cytoplasm, it is present in an inactive complex form coupled to the inhibitory protein IKB. It can be activated through a classical (canonical) or alternative (noncanonical) pathway [36]. The canonical NF-κB pathway is mediated by a NEMO-dependent IKK and is activated by proinflammatory signals, such as danger-associated molecular patterns (DAMPs), cytokines, and pathogen-associated molecular patterns (PAMPs). The activation by these agents leads to the activation of NEMO-containing kinase complexes, the phosphorylation and activation of IκB-kinase (IKK) that phosphorylates IκB (IκBα, IκBβ, IκBε). The phosphorylation of IκB induces its degradation and ubiquitination in the proteasome, which leads to the translocation of NF-κB into the nucleus. NF-κB then binds to DNA control elements and activates gene expression [36]. Besides, the noncanonical pathway is mediated by a NEMO-independent IKK1 kinase complex and is activated through a cascade of signals that activate NIK/IKK1 and result in the degradation of Iκbδ and the translocation of RelB: p52, RelB: p50, and RelA: p50 dimers to the nucleus to regulate gene expression [36]. ROS can modulate the expression of genes that promote carcinogenesis by altering the NF-κB signaling pathway. They can regulate the NF-κB pathway, both positively or negatively. They inactivate NF-kB function directly through the oxidation of IKKβ on cysteine 179, or the oxidation of p50 on cysteine 62 located in RHD, and, therefore, inhibit NF-κB binding to DNA; or indirectly through the deregulation of other proteins involved in NF-κB expression. For instance, ROS can activate the phosphorylation of RelA on Ser-276 by PKAc, which activates NF-κB [37]. H_2_O_2_ can also activate NF-κB through spleen tyrosine kinase (Syk)-mediated tyrosine phosphorylation of IκBα [38].

### 3.4. ROS and Phosphoinositide 3 Kinases (PI3K)/Akt Signaling Pathway

PI3Ks, a group of plasma-membrane-associated lipid kinases, contains two regulatory subunits (p85 and p55) and a catalytic subunit (p110) [39]. They catalyze the conversion of phosphatidylinositol-4,5-biphosphate (PIP2) to phosphatidylinositol-3,4,5-triphosphate (PIP3), which allows the phosphorylation of Akt through phosphoinositide-dependent protein kinase (PDK1) and the activation of its downstream signaling. PI3K/Akt signaling is negatively regulated by the tumor suppressor gene PTEN, which converts PIP3 into PIP2 and reduces cell proliferation and migration [40]. Oxidative stress mediates PI3K/Akt signaling activation directly through the inhibition of phosphatases, such as PTEN, or indirectly through the activation of Akt by other proteins. PTEN activity is highly affected by redox state [40,41]. It can be inactivated directly through the oxidation of its cysteine residues, which enhance the expression levels of PIP3 and activate AKT signaling and the expression of genes involved in cell survival [42]. In addition, ROS can indirectly activate Akt through an EGFR/PI3K-dependent pathway [43].

### 3.5. ROS and Hypoxia-Inducible Factor 1 (HIF-1) Signaling in Cancer

HIF-1, a heterodimeric transcription factor, mediates the response to hypoxia. It controls the expression of different genes implicated in apoptosis, cell cycle, cellular metabolism, and angiogenesis. It is overexpressed in various ROS-induced cancers, such as breast, ovarian, and prostate cancer. The elevated HIF-1 expression is induced and stabilized by ROS in hypoxic tumors [44]. It was demonstrated that high expression of epidermal growth factor (EGR) in ovarian cancer induces H₂O₂ production, which activates PI3K/Akt/p70S6K1. Activated p70S6K1 stimulates HIF-1a expression and, consequently, increases the expression of VEGF that mediates angiogenesis [45].

## 4. Oxidative-Stress-Induced Epigenetic Instability in Cancer

Cancer is one of the major health issues around the globe. It occurs due to various genetic and epigenetic alterations. These alterations occur as a result of various internal factors (e.g., DNA polymerase errors, oxidative stress, spontaneous deamination, etc.) [32,46] and external factors (e.g., stress, radiation, microbes, etc.) [47,48]. Genetic alterations, including chromosomal aberrations, amplification, mutations, deletions, insertions, etc., deregulate the expression of tumor suppressor genes (TSGs), DNA repair genes, and oncogenes, which lead to abnormal cell growth. Epigenetic refers to the modification of gene expression and chromatin structure without any change in DNA sequence. It is a crucial mechanism that ensures many physiological processes, including gene imprinting, differentiation, reproduction, cell division, etc. However, a deregulation of this mechanism is associated with different pathologies. Epigenetic instability is one of the key characteristics of cancer. It involves the alteration of chromatin remodeling, the deregulation of noncoding RNAs (ncRNAs), and histone modifications, as well as the modification of DNA methylation status [49]. These modifications alter gene expression, replication, and DNA repair.

Oxidative stress is one of the crucial factors in cancer development. It can induce genetic damage or alter epigenetic mechanisms. It can also induce various types of DNA damage and modifications in DNA structure, including deletions, strand breaks, chromosomal aberrations, base and sugar lesions, DNA–protein cross-links, etc. Furthermore, it can induce alterations to the cancer epigenome, including DNA methylation, deregulation of lncRNA expression, and modification of histones and chromatin remodelers [50]. Furthermore, oxidative stress can oxidize proteins and lipids and generate other reactive products that can damage DNA [50].

### 4.1. Oxidative Stress and DNA Methylation

DNA methylation is an essential gene regulation mechanism that causes transcription repression. It occurs in sequences with a high frequency of CG dinucleotide repeats, known as CpG islands, which can be found in the promoter or non-promoter regions. Different enzymes mediate DNA methylation, including DNA methyltransferases (DNMTs), methyl-CpG binding proteins (MBPs), and methyl CpG binding protein 2 (MeCP2). DNMTs form 5-methylcytosine (5mC) by adding a methyl group to the 5′ carbon of cytosine nucleotides. MBPs and MeCP2 have the capacity to discriminate between methylated and unmethylated CpG dinucleotides. They bind to methylated CpG sites and recruit chromatin remodelers and histone deacetylases (HDACs) that remove the acetyl group from acetylated lysine residues of histone proteins and inactivate gene expression [51,52]. Hypermethylation of CpG islands located on tumor suppressor gene promoters (TSGs) or DNA repair gene promoters induces inactivation of their expression [53]. In contrast, hypo- or demethylation of CpG islands that are located on oncogene promoters or non-promoter regions of repetitive elements, transposable elements, and retroviruses induces an abnormal activation of gene expression and genome instability [54].

Oxidative stress induces the oxidation of different oligonucleotides, including guanine, thymine, and 5 mC. The oxidation of guanine to 8-oxoguanine (8-oxoG) represents the major form of DNA damage, and is considered a biomarker of oxidative damage. This oxidation replaces the 8-proton with an oxygen atom and converts the N7 position of the guanine from an H-bond acceptor to an H-bond donor, which can inhibit the methylation of the cytosine residue adjacent to that of 8-oxoG residue [55]. Furthermore, 8-oxoG has a mutagenic potential, since it allows the misincorporation of deoxyadenosine-5′-monophosphate (dAMP) and dCMP during replication and allows the generation of GC–AT transversion mutations [56,57]. Additionally, ROS can also generate *O⁶*-methylguanine that inhibits methylation of the adjacent cytosine by inhibiting the binding of methyltransferases and, therefore, induces hypomethylation [58]. Moreover, the 5-methyl group of 5 mC allows MBPs to discriminate between methylated and unmethylated DNA. However, its hydroxylation can generate 5-formylcytosine, 5-hydroxymethylcytosine (HmC), or 5-carboxycytosine, which inhibit the binding of MBPs and alter epigenetic mechanisms. In addition, oxidation of the methyl group of thymine generates 5-hydroxymethyluracil (HmU), which interferes with transcription factor binding [55].

ROS can also alter the expression of proteins by increasing the expression of chromatin remodelers involved in DNA methylation. For instance, ROS increases the expression of Snail, a methyl-CpG binding protein that recruits DNA methyltransferase 1 (DNMT1) and histone deacetylase 1 (HDAC1), and induces the hypermethylation of E-cadherin, a tumor suppressor gene. The downregulation of E-cadherin expression is correlated with epithelial-to-mesenchymal transition, metastasis, tumor progression, and poor prognosis in hepatocellular carcinoma [59]. In addition, the increased expression of DNMT1 and HDAC1 induced by ROS can inhibit the expression of different tumor suppressor genes, such as *caudal-related homeobox-1* (*CDX1*), that are related to the progression of colorectal cancer [60]. Furthermore, studies have shown that oxidative stress can mediate the hypermethylation and inactivation of the tumor suppressor gene *p16INK4A*, which enhances the malignant progression of renal cell carcinomas (RCCs) [61].

### 4.2. Oxidative Stress and Histone Modification

Histones are crucial structural proteins that ensure DNA packaging in the nucleus, regulate the accessibility of DNA to transcription factors, restriction enzymes, etc., and modulate transcription, replication, and repair [62,63]. They undergo different modifications, commonly acetylation, deacetylation, methylation, and demethylation. Different enzymes, such as histone acetyltransferases (HAT), bromodomain and extra terminal domain (BET) proteins, and histone deacetylases (HDAC), regulate histone acetylation and deacetylation. HATs catalyze the transfer of an acetyl group from acetyl-CoA to the ε-amino group of lysine residues at histone proteins, which reduce the affinity of histones to DNA, release the chromatin, and activate DNA transcription. In contrast, BET proteins (BRD2/3 and 4) recognize acetylated histone lysine and recruit HDACs that remove the acetyl group from lysine and increase the affinity of histones to DNA, and, hence, compact the chromatin and repress DNA transcription [62,64]. In addition, different enzymes regulate histone methylation and demethylation, such as histone demethylases (HDMs) and histone methyltransferases (HMTs). HMTs transfer the methyl group from S-adenosyl methionine (SAM) to arginine (R) and lysine (K) residues. In contrast, HDMs remove the methyl group from R and K residues of histones.

Oxidative stress can modify the acetylation and methylation status of histone molecules by altering the enzymes that are involved in chromatin remodeling. Hydrogen peroxide can induce the delocalization of a silencing complex containing DNMT1, DNMT3B, and members of polycomb repressive complex 4 (PRC4) from non-GC-rich to GC-rich areas. This complex induces different changes in histone marks. For instance, SIRT1, a member of PRC4 reduces histone active marks, such as 3MeK4H3 and AcK16H4. PcG component of EZH2 activates the expression of histone repressive marks, such as H3K27me3. This complex can also increase DNA methylation and gene silencing in colon cancer [65].

### 4.3. Oxidative Stress and miRNAs

MicroRNAs (miRNAs) are tiny noncoding RNAs measuring about 18 to 25 nucleotides. They have a major role in the post-transcriptional regulation of gene expression [66] and they are involved in many physiological mechanisms, including apoptosis, cell cycle regulation, differentiation, proliferation, development, stress response, etc. [67]. They are located at different DNA regions, including noncoding genomic regions, intronic or exonic regions of coding genes [67], fragile sites, common breakpoint regions, minimal regions of amplification, and minimal regions of loss of heterozygosity [68]. miRNAs are transcribed in the nucleus to primary miRNAs (pi-miRNAs) by RNA polymerase II or III. Pi-miRNAs are then polyadenylated, capped, and cleaved in the nucleus by a complex involving drosha ribonuclease and DGGR8, forming hairpins called precursor miRNAs that are exported by exporting-5 to the cytoplasm. Once in the cytoplasm, a ribonuclease Dicer cleaves the pre-miRNA and generates a double-stranded RNA molecule. One of the strands is degraded, while the second one is stably associated with RNA-induced silencing complex (RISC), and it binds to a homologous 3′ untranslated region (3′-UTR) of an mRNA in order to block its translation or favor its degradation [69].

The levels of miRNA in tumor cells are altered, and oxidative stress is one of the factors that contributes to this alteration. Oxidative stress can influence miRNA expression by altering the expression of enzymes involved in miRNA biogenesis, inducing epigenetic changes, such as methylation, or directly damaging miRNA genes or mature sequences [70]. For instance, ROS can inhibit *miR-199α* and *miR-125b* gene expression in ovarian cancer by increasing hypermethylation of their promoters via DNMT1. The low expression of these miRNAs induces overexpression of the epidermal growth factor receptor family, including ERBB2/3, that is associated with cancer development, poor prognosis, and drug resistance [71]. In addition, ROS promote gastric cancer by upregulating the expression of miR-21, which subsequently downregulates the expression of programmed cell death 4 protein (PDCD4), a tumor suppressor gene that inhibits tumor progression and neoplastic transformation. The inhibition of this gene increases metastasis, tumor invasion, and cell transformation [72]. ROS also induce overexpression of miR-500a-5p and decreased expression of thioredoxin reductase 1 (TXNRD1) and nuclear factor, erythroid-2-like 2 (NFE2L2) that are involved in cellular antioxidant response. This deregulation is associated with poor prognosis in breast cancer [73]. Similarly, miRNA also regulates ROS production in cancer by targeting proteins implicated in the production or elimination of ROS [70]. A study has demonstrated that *miR-155* is upregulated by the oncogene K-Ras through MAPK–AP1 and NF-kB pathways in pancreatic cancer. This upregulation promotes ROS levels through the inhibition of *Foxo3a* expression, which leads to decreased expression levels of *SOD2* and catalase antioxidants and allows for tumor progression [74].

## 5. Antioxidant Protective Effects of Natural Compounds

### 5.1. In Vivo Preclinical Investigations

#### 5.1.1. Flavonoids

The antioxidant activity of flavonoids (Table 1) was described in numerous works using several in vivo models [75,76,77,78,79,80,81,82,83,84,85,86,87,88,89,90,91]. Indeed, Yue et al. [91] investigated the protective effect of apigenin against CCl_4_-induced hepatotoxicity in mice. The results showed that this flavonoid significantly protected liver tissue by elevating the SOD, GSH, GSH-Px, and CAT levels, and reducing MDA. The potential of this compound was tested in streptozotocin-induced diabetic mice in in vivo studies conducted by Mao et al. [82] and Liu et al. [81]. The results demonstrated that apigenin suppressed oxidative stress by various mechanisms, such as reducing the malondialdehyde content and increasing GSH and SOD levels.

The study of Demir et al. [75] found that oral administration of catechin 20 mg/kg ameliorated chlorpyrifos-induced oxidative stress in rat erythrocytes by the reduced malondialdehyde levels and elevated SOD, CAT, and GPx activities. The mechanism by which this compound elevated the levels of enzymatic and nonenzymatic antioxidant activities may involve the dismutation of O_2_.- and the decomposition of hydrogen peroxide, and may represent an aspect of the cellular response to increasing the levels of reactive oxygen species. On the other hand, oral administration of 50 mg/kg attenuated inappropriate alterations of the oxidative stress that was induced by STZ in mice by reducing malondialdehyde content, ROS, and protein carbonyl as a marker of lipid peroxidation and enhancing antioxidants, including SOD, CAT, and GPx activities [86]. Recently, Wan et al. [88] reported that injection of acetaminophen in mice induced acute liver injury by the formation of ROS and the perturbation of cellular antioxidant defense. Moreover, hesperetin pretreatment attenuated this oxidative stress, which was indicated by increasing antioxidative molecule production (GSH, SOD, and CAT) and decreased MDA levels. The antioxidant activity of this flavonoid may be due to neutralizing ROS and strengthening the defense system of the cell, including ERK/Nrf2 signaling.

Quercetin has been known as an interesting antioxidant compound. Its antioxidant efficacy was demonstrated in streptozotocin-induced diabetic rats, and chlorpyrifos- and NaN_3_-induced oxidative stress in rats. The data from these studies showed that quercetin decreased MDA levels [75,87], increased sperm SOD, CAT, and GPx activities and their mRNA expression levels [85,90], and elevated GSH, GSH-Px, and GR activity [80].

Rutin also showed a potent antioxidant effect. La Casa and collaborators [79] studied the gastroprotective effect of this compound using ethanol (50%) to induce gastric lesions. They reported that rutin at a concentration of 200 mg/kg has a protective activity. It decreased the gastric mucosal damage and elevated the antioxidant enzymatic glutathione peroxidase. At the same time, our results revealed an anti-lipoperoxidant effect via decreasing the levels of TBA [79]. In addition to attenuating oxidative stress, rutin downregulated endoplasmic reticulum stress markers GRP78 and CHOP in alloxan-induced diabetic nephropathy [76].

The antioxidant effect of resveratrol was also evaluated in vivo using ethanol [92], doxorubicin [93], lipopolysaccharide [94], and hyperglycemia [95] induced oxidative stress in mice. The results of different works reported that this natural compound exhibited potent antioxidant activity via several mechanisms, including the decrease in the content of malondialdehyde, promoting the activities of SOD, CAT, and GSH-Px. It also modulates the SIRT1/FOXO3a pathway by elevating SIRT1deacetylase activity.

#### 5.1.2. Phenolic Acids

Numerous reports have evaluated the antioxidant activities of phenolic acids (Table 2) [97,98,99,100,101,102,103,104,105,106,107,108]. In a study reported by Tolba et al. [107], caffeic acid showed a potential antioxidant activity by enhancing glutathione peroxidase content and SOD activity and significantly decreasing femur bone MDA in glucocorticoid-induced osteoporosis in vivo. Shen and collaborators studied the antioxidant effect of p-coumaric acid on a high-fat diet (HFD) mice model. They found that p-coumaric acid can increase the expression of Nrf2, Gpx, SOD-1, and HO-1. In addition to these effects, p-CA elevated CAT level in serum, total antioxidant capacity, and GSH-Px levels in the liver, and ameliorated lipid peroxidation [104].

Ferulic acid is another phenolic acid that exhibits a protective activity against cisplatin-, acetaminophen-, and formaldehyde-induced oxidative stress. The authors showed that oral administration of ferulic acid inhibited the expression of cytochrome P450 2E1, increased SOD, GPx, and CAT activities, and GSH levels. It also protected lipid peroxidation by decreasing MDA levels [100,102,108].

In another work, Nabavi et al. [103] investigated the antioxidant effect of gallic acid in vivo by determination of the SOD and CAT activities, TBARS, and GSH levels for mouse brains. They found that oral administration of GA improved the antioxidant system by increasing SOD and CAT activities and GSH content, as well as decreasing TBARS levels. Govindaraj and Sorimuthu Pillai [101] studied the protective effect of rosmarinic acid on rats with a high-fat diet and streptozotocin-induced diabetic and oxidative stress, and the results demonstrated that this phenolic acid decreased the lipid peroxide, AOPP, and protein carbonyl levels, and increased enzymatic antioxidant (superoxide dismutase and catalase) and nonenzymic antioxidants (GPx, GSH, Vit. C, and Vit. E) in diabetic rats [101]. This result was confirmed by Baranauskaite et al. [97] and Thingore et al. [106], which proved a decrease in MDA concentration and an increase in SOD and CAT activities after treatment with rosmarinic acid. On the other hand, the study of Dianat and their colleagues demonstrated that vanillic acid reduced the malondialdehyde content, as well as elevated SOD, CAT, and GPx activities on ischemia-reperfusion-induced oxidative stress in isolated rat hearts [99].

#### 5.1.3. Terpenoids

Terpenoids are volatile compounds that have various biological proprieties, such as antioxidant activity. Indeed, more than 20 works (Table 3) studied this effect in vivo [92,93,94,95,96,97,109,110,111,112,113,114,115,116,117,118,119,120,121,122,123,124,125,126].

Shata and Eldebaky [118] evaluated the antioxidant activity of camphor oil against atrazine-induced oxidative stress in vivo. They found that camphor treatment at a concentration of 30 mg/kg showed antioxidant properties by the reduction in MDA levels and a significantly elevated activity of SOD. Rajan et al. [116] induced liver damage by N-nitrosodiethylamine in experimental rats and measured lipid peroxidation and the antioxidant status. The authors noted that this monoterpene attenuates oxidative damage by increasing host enzymatic antioxidants (superoxide dismutase, catalase) and nonenzymic antioxidants (GPx, GR, GSH, G6PD, Vit. A, Vit. C, and Vit. E). This result was validated recently by Baranauskaite et al. [97], who reported that the daily administration of 0.0405 mg/kg of camphor protects the brain and liver against oxidative stress in rats via activation of the antioxidant defense system. On the other hand, the study of Mishra et al. [114] showed that treatment with citral at a concentration of 45 mg/kg to streptozotocin/high-fat-diet-induced diabetic dyslipidemic rats ameliorated the activity of SOD, catalase, GPx, and GR, and also increased GSH content in all tissues. It reduced lipid peroxidation and protein carbonyl content. Recently, Bagheri and collaborators investigated the antioxidant effects of D-limonene in alloxan-induced diabetic rats. They revealed that limonene treatment at a dose of 100 mg/dL significantly decreased serum MDA, MPO, and NO, while increasing GSH. This compound was also found to elevate GPx, CAT, and SOD levels in mRNA [110].

Geraniol is another terpenoids compound that showed antioxidant activity in vivo. In a study by Younis et al. [125], geraniol revealed a hepatoprotective effect in liver ischemia reperfusion injury induced by oxidative stress. This effect was associated with various molecular mechanisms, such as increasing the TAC and GSH levels, decreasing MDA levels, activation of Nrf2, as well as the upregulation of HO-1 expression. In the same years, El-Emam et al. [111] confirmed this result. The authors reported that oral administration of geraniol 100 and 200 mg/kg exhibited a protective effect against isoproterenol-induced cardiotoxicity by decreasing Keap1 expression and increasing nuclear accumulation of Nrf2

Kaempferol was tested against alcoholic liver injury in mice. It attenuated the activity and expression of CYP2E1 and reduced oxidative stress, as well as lipid peroxidation, and elevated the antioxidative system [121]. Moreover, the oral administration of linalool at a dose of 100 mg/kg reduced the AChE level, which has a close relationship with oxidative stress, and increased SOD and GPX, as well as activated Nrf2/HO-1 signaling [123].

Agarwal et al. [109] investigated the protective activity of thymol against β-cell damage induced by streptozotocin. This effect was concluded by assessing plasma MDA content, advanced oxidation protein products (AOPP), and sialic acid (SA). Markers of antioxidants, such as FRAP and GSH, were also measured. The results of the study demonstrated that giving thymol with a dose of 40 mg/kg reduced the MDA level, sialic acid and AOPP levels, and also elevated % DPPH, FRAP content, and erythrocyte GSH levels.

### 5.2. Clinical Evidence

Clinical evidence of the natural antioxidant compound is a very crucial step for the development of a new antioxidant drug. Indeed, several studies have investigated this effect in patients or healthy individuals, and the results are presented in Table 4, which reports the compound names, the human subjects, and major results [127,128,129,130,131,132,133,134,135]. Agarwal et al. [127] examined the effect of catechin at a concentration of 500 μg on biomarkers of oxidative stress in patients diagnosed with AFB-positive cases of pulmonary tuberculosis. Accordingly, catechin significantly decreased lipid peroxidation and NO production levels. It also decreased catalase, GPx, and SH levels, while increasing SOD and GSH levels.

Furthermore, De Groote and collaborators [131] demonstrated that catechin and resveratrol showed a protective effect against oxidative stress in obese subjects by numerous mechanisms, including the increase in the activity of GPX, SOD, TAP, and GSSG, a decrease in some oxidative damages, and modulation of the expression of genes involved in redox and cellular stress response pathways. In another study performed in 2014 on healthy postmenopausal women, acute ingestion of catechin had no substantial effect on the levels of postprandial oxidative stress markers, but increased postprandial TRX concentrations, which has a vital role in cellular function and protection by limiting oxidative stress directly via its antioxidant capacity [135]. Regarding quercetin, a study by Quindry et al. [134] investigated the antioxidant activity of quercetin supplementation against blood oxidative stress in athletes during ultramarathon competition. The authors showed that quercetin supplementation increased antioxidant capacity and plasma urate levels, while Trolox-equivalent antioxidant capacity remained unaffected, as well as plasma F2-isoprostane values and plasma protein carbonyls (biomarkers of plasma oxidative damage) in athletes. In another work, Boots et al. [129] revealed that quercetin supplementation improved the antioxidant system by improving TAC and decreasing MDA concentration. This effect was confirmed by Duranti et al. [132], who demonstrated that this flavonoid decreased erythrocytes lipid peroxidation levels and improved redox status in healthy young after a strenuous eccentric exercise. Bumrungpert et al. [130] assessed the antioxidant activity of ferulic acid (1000 mg daily) in patients with hyperlipidemia by determination of biomarkers of oxidative stress and reported that ferulic acid supplementation improved oxidative stress by decreasing the MDA content (24.5%). In another work, Ferk et al. [133] investigated the preventive activity of gallic acid (15 mg/p/d) against oxidative stress in diabetic patients. Accordingly, gallic acid consumption did not affect MDA concentration, which is formed as a consequence of lipid peroxidation, as well as FRAP, which reflects the overall redox status, while protecting the genetic material against oxidative damage in the diabetic patients. In a clinical study by Alavinezhad et al. [128], asthmatic patients received carvacrol at a concentration of 1.2 mg/kg/day and reported a decrease in the plasma level of NO^2-^.

## 6. Conclusions

The use of natural antioxidants specifically targeting ROS generation will allow cellular oxidative stress to be avoided, and, consequently, minimize the epigenetic instability, which induces the first events of tumor transformation. Indeed, we have reported that natural antioxidant molecules have proven their effectiveness in vivo, as well as clinically, in the inhibition of oxidative stress (the effectors of stress and its generation). This demonstrates that these substances could build major molecular building blocks in the screening of antioxidant substances used in the prevention of human cancers. However, the molecular action of these substances on the generation of free radicals remains to be determined in future investigations. In addition, the action of these molecules should also be investigated in epigenetic modifiers to prevent epigenetic instability and tumor transformation. The clinical applications of these molecules as antioxidant drugs against oxidative-stress-related diseases, such as cancer, need further investigation. Indeed, toxicological studies are needed to validate the safety of these bioactive compounds. In addition, pharmacokinetic investigations should also be carried out to determine the distribution, metabolism, and elimination of these drugs. On the other hand, in our opinion, these substances should be also tested as epidrugs (in vivo and clinically) on different mediators of epigenetic modifications induced by oxidative stress. In this way, drugs that target oxidative-stress-inducing cancer via epigenetic-instability- and epigenetic-dysregulation-induced cell transformation could be screened and used in pharmaceutical applications to prevent and treat human cancers.

## Figures and Tables

**Table 1:** In vivo antioxidant activity of flavonoids.

Compounds	Experimental Approach	Key Results	Refs
Apigenin	CCl4-induced hepatotoxicity in mice	SOD, CAT, GSH-Px, and GSH levels increased.	[91]
		MDA level was decreased.	
Apigenin	Kainic acid (KA)-induced excitotoxicity	GSH levels were increased.	[78]
Apigenin	STZ-induced diabetic cardiomyopathy	SOD and GPx activity were increased.	[81]
		Decreased GSH levels.	
Apigenin	H_2_O_2_-induced rat hepatic stellate cells	SOD and GSH levels were enhanced.	[83]
		ROS, MDA, and NO levels were inhibited.	
Apigenin	Diabetes-associated cognitive decline a diabetic rat model	Decreased the MDA content.	[82]
		Increased SOD activity and GSH level.	
		Inhibited the activities of cNOS and iNOS.	
Apigenin	Myocardial ischemia/reperfusion injury in mice	Significantly decreased MDA.	
		Elevated SOD activity.	
Catechin	Subacute chlorpyrifos-induced oxidative stress	Reduced MDA content.	[75]
		SOD, CAT, and GPx activities were increased.	
Catechin	Type 2 diabetic erythrocytes	Decreased MDA.	[84]
		Increased GSH.	
Hesperetin	Acetaminophen-induced hepatotoxicity	Increased levels of glutathione. Increased SOD and CAT activities.	[88]
		Reduced MDA levels.	
Hesperetin	Streptozotocin-induced diabetic in rat	Increased GSH.	[86]
		Improved CAT, SOD, and GPx.	
		Decreased levels of MDA.	
		Reduced protein carbonyl.	
Quercetin	Subacute-chlorpyrifos-induced oxidative stress	Decreased malondialdehyde levels.	[75]
		Enhanced SOD, CAT, and GPx.	
Quercetin	Streptozotocin-nicotinamide-induced diabetic rats	Improved SOD, CAT, GPx.	[90]
		Increased mRNA expression levels.	
		Ameliorated MDA levels.	
Quercetin	Streptozotocin-nicotinamide-induced diabetic rats	Improved cardiac SOD-1, CAT, and GPx-1.	[85]
Quercetin	Myocardial ischemia reperfusion (IR) injuries	Reduced MDA content.	[80]
		Increased the activities of GSH, SOD, CAT, GSH-Px, GR.	
Quercetin	Sodium-azide-induced hepatic and splenic oxidative stress in vivo	SOD and GPx activities were significantly increased.	[87]
		Considerably reduced MDA concentrations.	
Rutin	Intestinal toxicity induced by methotrexate	Decreased TBARS and protein carbonyl.	[77]
		Increased SOD, catalase, and GSH.	
Rutin	Alloxan-induced diabetic nephropathy	Increased SOD and catalase.	[76]
		Reduced lipid peroxidation.	
		Downregulated endoplasmic reticulum stress markers GRP78 and CHOP.	
Rutin	Gastric lesions induced by 50% ethanol	Significantly increased GSH-Px activity.	[79]
		Decreased the levels of thiobarbituric acid.	
Resveratrol	Ethanol-induced oxidative stress in vivo	Increased SOD activity.	[92]
		Increased catalase.	
		Increased glutathione peroxidase.	
Resveratrol	Oxidative stress cardiomyopathy induced by doxorubicin	Reduced MDA content.	[93]
		Promoted SOD, CAT, and GPx activities.	
		Increased GSH.	
Resveratrol	Lipopolysaccharide-induced oxidative stress	Significantly reduced the level of TBARS.	[94]
		Significantly increased glutathione level and the superoxide dismutase.	
Resveratrol	Hyperglycemia-induced renal tubular oxidative stress damage	Prevented the SOD activity downregulation and MDA upregulation.	[95]
		Significantly increased CAT levels.	
		Modulates the SIRT1/FOXO3a pathway.	
Resveratrol	Murine model of lipopolysaccharide (LPS)-induced pulmonary fibrosis	Decreased MDA levels.	[96]
		Increased total antioxidant activity, superoxide dismutase, and catalase activities.	

**Table 2:** In vivo antioxidant activity of phenolic acid.

Compounds	Experimental Approach	Key Results	Refs
Caffeic acid	Glucocorticoid-induced osteoporosis in vivo	Elevated glutathione peroxidase content and superoxide dismutase.	[107]
		Significantly decreased malondialdehyde levels.	
Caffeic acid	Medium-term rat hepatocarcinogenesis model	Decreased lipid peroxidation.	[98]
p-Coumaric acid	High-fat diet (HFD) mice model	Elevated CAT, total antioxidant capacity, and GSH-Px levels.	[104]
Ferulic acid	Cisplatin-induced ototoxicity	Increased SOD and GPx activities.	[100,102,108]
		Reduced MDA levels.	
Ferulic acid	Acetaminophen-induced hepatotoxicity	Enhanced superoxide dismutase and catalase activities.	[108]
		Increased GSH-Px levels.	
Ferulic acid	Formaldehyde-induced hepatotoxicity	Increased CAT, GPx content, and SOD activities.	[100]
		Decreased malondialdehyde content.	
Gallic acid	Balb/c mice with post-stroke depression	Increased SOD and CAT activities.	[103]
		Elevated glutathione peroxidase content.	
		Decreased TBARS levels.	
Gallic acid	Cerebral ischemia/reperfusion-induced by middle cerebral artery occlusion	Reduced MDA levels.	[105]
Rosmarinic acid	Aluminum-induced oxidative stress	Increased GSH concentration.	[97]
		Decreased MDA concentration.	
		Increased CAT and SOD activities.	
Rosmarinic acid	High-fat diet and streptozotocin-induced diabetic rats.	Elevated the levels of vitamin C, vitamin E, and GSH.	[101]
		Elevated SOD, CAT, and GPx activities.	
		Decreased lipid peroxide, AOPP, and protein carbonyl levels.	
Rosmarinic acid	Lipopolysaccharide-induced memory impairment	SOD activity increased.	[106]
		GSH levels reduced.	
		Decreased lipid peroxidation in the brain.	
Vanillic acid	Ischemia-reperfusion-induced oxidative stress in isolated rat heart	Decreased MDA.	[99]
		Elevated SOD, CAT, and GPx activities.	

**Table 3:** In vivo antioxidant activity of terpenoids.

Compounds	Experimental Approach	Key Results	Refs
Camphor	Atrazine-induced toxicity	Increased SOD activity.	[118]
		Reduced MDA levels.	
Carvacrol	N-nitrosodiethylamine-induced liver injury in mice	Decreased the levels of lipid peroxides.	[116]
		Elevated superoxide dismutase and catalase activities.	
		Significantly increased the activities of GPx, GR, GSH, G6PD, vitamin (Vit. A), Vit. C and Vit. E.	
Carvacrol	Acute myocardial infarction	Decreased MDA content.	[126]
	in mice	Increased SOD, GSH, and GSH-Px activities.	
Carvacrol	Restraint-stress-induced oxidative stress damage in the brain, liver, and kidney	Reduced MDA content.	[117]
		Elevated GSH, SOD, GPx, GR, and CAT activities.	
Carvacrol	Alloxan-induced diabetic rats	Reduced malondialdehyde.	[112]
		Increased significantly glutathione levels.	
Carvacrol	STZ-induced diabetic rats	Reduced levels of tissue malondialdehyde.	[119]
		Increased antioxidant enzymes (SOD and GPx,).	
Carvacrol	Weaning-induced intestinal dysfunction in piglets	Significantly elevated superoxide dismutase and glutathione peroxidase activities.	[122]
		Decreased TBARS levels.	
Carvacrol	Aluminium-induced oxidative stress	Increased GSH concentration.	[97]
		Decreased MDA concentration.	
		Increased CAT and SOD activities.	
Citral	Streptozotocin/high-fat-diet-induced diabetic dyslipidemic rats	Significant reduction in the level of MDA.	[114]
		Attenuated protein carbonyl content.	
		Significantly improved the activity of SOD.	
		Significantly restored the activity of catalase.	
		Significant increase in Gpx activity.	
D-limonene	Alloxan-induced diabetic rats	Reduced malondialdehyde and NO.	[110]
		Elevated GSH levels.	
		Increased GPx, CAT, and SOD activities.	
		Significant elevation in mRNA levels of superoxide dismutase, catalase, and glutathione peroxidase activities.	
Geraniol	Hepatic ischemia reperfusion injury	Increased GSH levels.	[111]
		Normalized malondialdehyde.	
		Decreased Keap1 expression.	
		Elevated the nuclear accumulation of Nrf2.	
		Elevated expression levels of HO-1.	
Geraniol	Isoproterenol-induced cardiotoxicity	Increased GSH levels.	[125]
		Elevated GPx, CAT, and SOD activities.	
		Activated Nrf2.	
		Upregulation of HO-1 expression.	
Kaempferol	Alcohol-induced liver injury in mice	Increased antioxidant enzymes (superoxide dismutase and glutathione).	[121]
		Decreased malondialdehyde.	
		Attenuating the activity and expression of CYP2E1.	
(-)-linalool	Oxygen–glucose-deprivation-induced neuronal injury	Significantly increased SOD.	[115]
Linalool	Amyloid-beta-induced cognitive deficits and damages in mice	Elevated dismutase and glutathione peroxidase activities.	[123]
		Reduced the malondialdehyde content.	
		Reduced the AChE level.	
		Activated the Nrf2/HO-1 signaling.	
Thymol	Lipopolysaccharide-induced acute lung injury mice	Decreased malondialdehyde and MPO levels.	[120]
	Model	Increased superoxide dismutase activity.	
Thymol	Type 2 diabetes in a streptozotocin-induced rat model	Significantly improved FRAP value.	[109]
		Decreased the levels of AOPP value.	
		Significantly decreased MDA level.	
		Elevated erythrocyte GSH levels.	
		Elevated % DPPH.	
Thymol	LPS-induced acute lung injury in mice	Significantly reduced the MPO activity.	[124]
		Significantly reduced MDA content.	

**Table 4:** Clinical evidence of antioxidant activity of natural compounds.

Molecules	Human Subjects	Effects	Refs.
Catechins	Patients of AFB-positive pulmonary tuberculosis.	Decreased MDA concentration.	[127]
		Decreased the level of NO production.	
		Significantly increased SOD.	
		Significantly decreased catalase and GPx level.	
		Significantly increased GSH.	
		Significant decrease in SH level.	
Catechin	Adult obese subjects	Increased the gene expression, and also SOD and GPX activity.	[131]
		Reduced the levels of GSH.	
		Increased TAP and GSSG.	
		Decreased lipid peroxides.	
		Altered the expression of genes involved in redox.	
Catechins	Healthy postmenopausal women	No impact on serum d-ROM concentrations and plasma H_2_O_2._	[135]
		Elevated postprandial plasma TRX concentrations.	
Quercetin	Athletes	Significantly increased plasma FRAP.	[134]
		Unaffected TEAC Plasma F2-isoprostane values and protein carbonyls.	
Quercetin	Non-smoking patients with symptomatic sarcoidosis	Increased total plasma antioxidant capacity.	[129]
		Decreased MDA concentration.	
Quercetin	Healthy young	Decrease in GSSG levels.	[132]
		Improved GSH/GSSG ratio.	
		Significantly decreased TBARs levels.	
		No effect in erythrocytes CAT, GPx, SOD activities, and SOD/GPx ratio.	
Ferulic acid	Subjects with hyperlipidemia	Decreased the oxidative stress biomarker, MDA.	[130]
Gallic acid	Type 2 diabetes patients	No impact on plasma MDA and FRAP.	[133]
Carvacrol	Asthmatic patients	Significantly decreased plasma level of NO^2^-.	[128]
Resveratrol	Adult obese subjects	Increased the gene expression, and also SOD and GPX activity.	[131]
		Reduced the levels of GSH and GSSG.	
		Increased TAP.	
		Decreased lipid peroxides.	
		Altered the expression of genes involved in redox.	
